# Visual Search Revived: The Slopes Are Not That Slippery: A Reply to Kristjansson (2015)

**DOI:** 10.1177/2041669516643244

**Published:** 2016-05-05

**Authors:** Jeremy M. Wolfe

**Affiliations:** Harvard Medical School, USA; Visual Attention Lab, Department of Surgery, Brigham & Women’s Hospital, USA

**Keywords:** visual search, reaction times, response times, serial search, parallel search, guided search, visual attention, selective attention

## Abstract

Kristjansson (2015) suggests that standard research methods in the study of visual search should be “reconsidered.” He reiterates a useful warning against treating reaction time x set size functions as simple metrics that can be used to label search tasks as “serial” or “parallel.” However, I argue that he goes too far with a broad attack on the use of slopes in the study of visual search. Used wisely, slopes do provide us with insight into the mechanisms of visual search.

Standard visual experiments ask observers to detect the presence or absence of a target item among some number of distractors. The total number of items in a display is the “set size.” Reaction times (RTs) and accuracy are measured and the slope of the RT × set size function is taken to give important insights into the nature of the underlying search. Arni Kristjansson (2015), in his piece, “Reconsidering Visual Search,” makes an important point about such experiments that apparently cannot be made too often. It is a mistake to take a measure of the slope of that RT × set size function and to declare, based on some criterion value of ms/item, that the underlying search is preattentive or parallel or attentive or serial. When I looked over a large body of work from my lab almost 20 years ago, I reported that search slope values form a continuum with no meaningful break between “parallel” and “serial” tasks (Wolfe, 1998). I argued for a theory-neutral description of slopes as indicating that searches were more or less “efficient.” Still, the notion of two stages—preattentive followed by attentive—lives on; along with the notion that some searches are done by the preattentive stage alone and others by the attentive stage. The origins of the idea arise with Neisser (1967) and become important as part of a theory of search with Treisman and Gelade’s (1980) “Feature Integration Theory.” It is sometimes held to imply that some pieces of brain anatomy are “preattentive” and others “attentive.” This is oversimplified. The field moved on years ago (for an updated look at Feature Integration Theory, see Treisman, 1998) but Kristjansson is quite right that this early, two-stage idea remains enshrined in textbooks and cited in work in neighboring fields.

Unfortunately, while it is worth reminding the field that there has been progress since 1980, Kristjansson (2015) wants to throw out a whole set of scientifically useful “babies” with this outdated, two-stage “bathwater.” The purpose of my short piece is to argue that the basic visual search paradigm continues to be useful and that RT × set size functions continue to be interpretable.

## Preattentive Processing Is Real

The idea of an autonomous “preattentive” piece of the visual system may be dangerous and wrong, but preattentive *processing* is a meaningful part of any theory of visual attention. If we assume that there is such a thing as visual selective attention, that means that some region or object is the current object of attention and that other regions and objects are not. When a new scene is presented to an observer, some regions and objects will not yet have been selected and, thus, will not have been subject to the effects of visual selective attention. If those objects are being processed at all—which, of course, they are—that processing is, tautologically, “preattentive.” If something is seen in regions that have not yet been attended—and, of course, something is seen there, then we can talk about “preattentive vision.” The nature of that preattentive processing and the contents of preattentive visual representations are open for investigation (Wolfe & Bennett, 1997) as is the relationship of preattentive to “postattentive” vision (Wolfe, Klempen, & Dahlen, 2000). However, if attention exists, the existence of the preattentive is not really open to question. It is possible that “preattentive” could be relabeled “weakly attended” on the assumption that some low level attention is always spread across the visual field, but that is a largely semantic distinction. If there is selective visual attention, then there are stimuli that have not yet been selected.

“Preattentive” is not a categorical label for a piece of the visual system. Neisser had a preattentive box in his original diagram but it is a mistake to think of that box as a dedicated preattentive piece of brain. If an item has not yet been attended, its representation is preattentive. Activity in, for example, V1—primary visual cortex—may be associated with that preattentive representation. However, a moment later, that item may be attended. Reentrant or feedback signals will modify the activity in V1 and the same piece of cortex will now be contributing to the attentive representation of the same item.

## RT × Set Size Functions Are Interpretable and Useful

**Figure 1.**
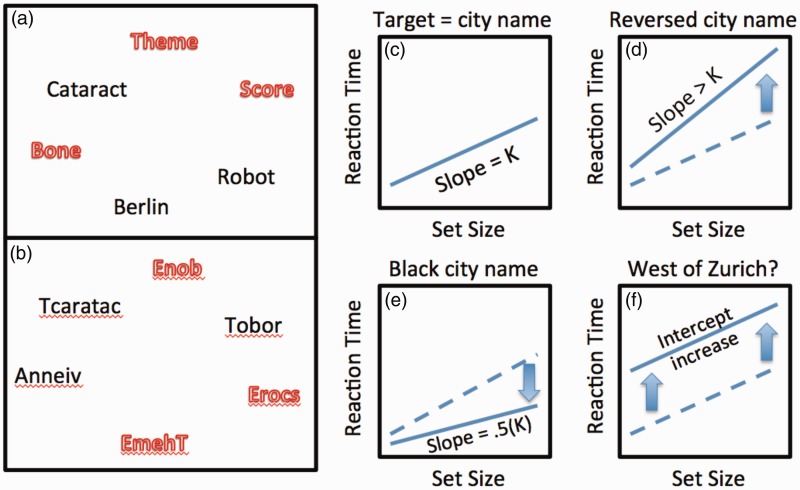
A simple search task: Find the name of a capital city. Letter order is reversed in 1(b). (c) to (f) cartoon results of manipulations of the basic experiment (see text).

Kristjansson’s (2015) main argument is that the slopes of RT × set size functions are ambiguous and that the RT methods pioneered by Donders (1868, 1969), Sternberg (1966), and Posner (1978) are not actually useful in the study of search. While there are complications and subtleties in the analysis of RT data in search, the basic logic of the analysis of RT × set size functions, like the basic logic of the idea of preattentive vision, seems quite solid. This can be illustrated with a toy example, shown in Figure 1.

In Figure 1(a), the task is to determine if one of these words names the capital of a country. You will find “Berlin” but, unless you got lucky, you will have had to read other words, one after the other, in series before getting to the target. If we did the task repeatedly, you would read, on average, half the words before stumbling on the target. If we varied the number of words in the display, we would vary the number of words you would need to read and, accordingly, the time required to find the target would grow linearly with set size, as cartooned in Figure 1(c). This would produce some slope of K ms/item. Now, suppose we reversed the order of the letters as in Figure 1(b). The task is the same and you can still find the capital, but you will need to spend markedly longer with each item. If each item takes longer, the result will be a steeper RT × set size slope (Slope > K, 1(d)). If you had been informed that the target word, if present, was written in black and not red (outline) letters, you still would have needed to search, but you would not have bothered to read red, outline words. Since only half the items are black, you would have read half as many words on average and, as shown by Egeth, Virzi, and Garbart (1984), the slope would be decreased by a factor of 2 (Slope = 0.5 K, 1(e)). This is the basic idea of feature guidance and the heart of the Guided Search model (Wolfe, 1994, 2007; Wolfe, Cave, & Franzel, 1989). If only one item was black on each trial, the slope would be zero because attention would go to the target word (when present) first time, every time. This very shallow slope would not indicate that word processing had become “parallel.” It would simply indicate perfect “guidance.” Slopes are an index of the amount of guidance and of the rate of processing of selected items (the two components can usually be teased apart with the right control experiments).

If the task was to determine if the target city lays to the east or west of Zurich, Switzerland, that would take longer. However, the added cost would not be imposed on each selected item, only on the target, once found. The result would be an intercept change (Slope = K, 1(f)). Changes to the nonsearch portion of a task will typically produce intercept changes.

This is a toy example and I have not collected real data. However, I would be happy to wager that the results will come out as advertised here if anyone cares to try the experiment. In this example, RT × Set Size functions really are interpretable. The slopes do not define a task as “serial” or “parallel” but they do carry meaning.

## But There Are Complications and Limitations

Of course, if life were this simple, there would be no controversy, but, as Kristjansson’s (2015) piece makes clear, there is controversy. The example in Figure 1 is made more straightforward by the choice of a task that virtually must have a serial component at its core. In this case, each word probably needs to be fixated before it is read, enforcing seriality. With tasks that do not require eye movements, models, like Guided Search, that propose covert, serial deployments of attention can be countered by models that propose parallel processing of all items (Palmer, 1995, see also Vincent, 2015). Standard RT × set size data will not distinguish these models (Townsend, 1990; Townsend & Wenger, 2004). Nevertheless, the empirical patterns from Figure 1 will remain intact. If you make processing of each item harder, slope increases. If you mark half the items as irrelevant with a salient feature, slope will be cut in half. In fact, search is neither “serial” nor “parallel.” Search RTs probably arise from a hybrid of serial and parallel processes (Wolfe, 2003). It is probably better to use RT × set size functions to ask “why is this search more (or less) efficient than that one?” and not to attempt to categorize a specific search as “serial” or “parallel.”

The empirical heart of the Kristjansson (2015) article lies in the differences he finds between results for the same search task produced by two different methods; a presence or absence version and a go or no-go version. Although interesting, the complications introduced here are not very troublesome to the RT × set size methodology. The go or no-go RTs are consistently faster. Different methods will often produce differences in mean RTs. For example, if Os need to fixate on a target to indicate that they have found it, they will typically have shorter RTs than if they need to move a mouse to the target. Negative slopes, of the sort seen in some of Kristjansson’s conditions, were a bit of a puzzle when first reported (Bravo & Nakayama, 1992) but these are typically understood as bottom-up salience effects. Negative slopes typically show up in “pop-out” searches (e.g., when the target is a salient color singleton). As the set size goes up, the density of stimuli goes up. As a result, on average, the target (e.g., a red item) will be surrounded more closely by dramatically different distractors (e.g., blue items). The local salience is defined by the relationship of an item to its neighbors, so a red item in a dense blue array is more salient than that item in a sparser display. If we assume that RT is a function of salience, RT will go down as set size and density increase (Santhi & Reeves, 2004; see Schoonveld, Shimozaki, & Eckstein, 2007, for an ideal observer account).

The features of the data in the Kristjansson paper that are potentially the most challenging are the changes in slope that occur when the only change is in the response made by the observer. For instance, in the Easy Conjunction condition, Kristjansson’s target-present slopes are near zero for the present or absent task while they are negative for the go or no-go task. Here, it is important to look at the error rates. The absolute error rates are not as important as the slope of the error rate × set size functions. Note that, for the two conjunction tasks, the go or no-go task produces a more positive error slope than the present or absent task. Higher errors tend to be associated with lower mean RTs on the correct trials (a classic speed-accuracy tradeoff). If the slope of the error function is positive, you get more depression of the RTs at the larger set sizes. This produces a shallower (or potentially negative) RT × set size slope. This is the pattern seen in the data in the Kristjansson (2015) paper. Such results require thought and competent researchers might differ about interpretation. However, while there is no reason to doubt the validity of the Kristjansson data, those data do not require wholesale abandonment of the RT × set size functions.

## In Sum …

Kristjansson performs a service when he warns against treating RT × set size functions as simple metrics that can be used to assign search tasks to the overly simple categories of “serial” and “parallel.” He goes too far with a sweeping attack on the utility of slopes in search tasks. With a bit of caution and a suitable set of experiments, slopes do provide us with insight about the mechanisms of visual search.
